# Prediction of air traffic delays: An agent-based model introducing refined parameter estimation methods

**DOI:** 10.1371/journal.pone.0249754

**Published:** 2021-04-07

**Authors:** Chunzheng Wang, Minghua Hu, Lei Yang, Zheng Zhao

**Affiliations:** 1 College of Civil Aviation, Nanjing University of Aeronautics and Astronautics, Nanjing, China; 2 National Key Laboratory of Air Traffic Flow Management, Nanjing University of Aeronautics and Astronautics, Nanjing, China; Huazhong University of Science and Technology, CHINA

## Abstract

We propose an agent-based model for predicting individual flight delays in an entire air traffic network. In contrast to previous work, more detailed parameter estimation methods were incorporated into the agent-based model, acting on the state transitions of agents. Specifically, a conditional probability model was proposed for modifying the expected departure time, which was used to indicate whether a flight had experienced the necessary waiting due to Ground Delay Programs (GDPs) or carrier-related reasons. Additionally, two random forest regression models were presented for estimating the turnaround time and the elapsed time of flight agents in the agent-based delay prediction model. The parameter models were trained and fitted using the flight data for 2017 in the United States. The performance of the delay prediction model was tested for thirty days with three types of delay levels (low, medium, and high), which were randomly selected from 2018. The experimental results showed that the average absolute error in the test days was 6.8 min, and the classification accuracy with a 15 min threshold for a two-hour forecast horizon was 89.5%. The performance of our model outperformed that of existing research. Additionally, the positive effect of introducing parameter models and the negative impact of increasing the prediction horizon on the prediction performance were further studied.

## Introduction

The possible flight delay time is becoming a significant reference for passengers when they plan their itineraries due to the frequency of flight delays. More than one-fifth of flights were delayed or cancelled between 2010 and 2018 in the United States of America. In particular, this value reached up to one-fourth in 2014. Flight delays not only interfere with the itineraries of passengers but also cause huge economic losses to the national economy. It is reported that flight delays have caused more than $32 billion in losses to individuals, societies, and airlines in 2007 in the United States of America [1]. In addition, flight delays have an unfriendly impact on the environment, which is mainly reflected in the occurrence of airborne delays that undoubtedly consume more fuel and increase the emissions of gases, such as carbon dioxide, accordingly [2].

The prediction of flight delays is challenging work because of the complexity of air traffic management (ATM) systems, which consist of many elements such as flights, airports, and airspace. The interactions of the various elements give rise to nonlinear aggregation [3,4]. Consequently, it is difficult to apply an analytical method to model the dynamics of system-wide delays. There are also a large number of factors that result in flight delays [5]. The demand and the capacity unbalancing of airspaces or airports originally caused most of the flight delays [6], which reduced the system robustness, resulting in flight delay propagation and even accumulation [7]. Moreover, the meteorological forecast is not always accurate [8], which may result in over-conservative or over-aggressive capacity estimation. The former may incur excessive ground holding, and the latter may result in expensive airborne delays [9]. With the exception of weather reasons, some random factors such as aircraft failures and human factors (a large number of human operators are involved in an ATM system) also increase the difficulty of prediction. Still, the economic losses caused by delays would be reduced to some extent by implementing effective air traffic management initiatives such as Ground Delay Programs (GDPs) if flight delay information can be accurately obtained in advance. Therefore, the important significance of flight delay prediction has prompted researchers to try various methods to achieve higher precision on a larger scale [10,11].

As an effective method for the analysis of dynamic behaviors of a complex system [12], simulation has been widely used for flight delay prediction. The National Airspace System Performance Analysis Capability (NASPAC), one of the first National Airspace System (NAS) simulation models, was developed by MITRE CAASD and used to analyze the performance of the National Airspace System [13]. Another NAS simulation tool, the Detailed Policy Assessment Tool (DPAT) was also developed by MITRE CAASD [14]. As a successor to NASPAC, there are some differences in enroute modeling, worldwide capability, and total execution time. In particular, for airport capacity, which is a key parameter for model input, DPAT applies a Pareto Frontiers method, which is also often used for airport capacity assessment. However, because of the limitation of the input to the aircraft itineraries, it fails to capture the propagation delay of flights in an ATS. Hence, two more fine-grained NAS simulation models [15,16] were developed. They are mainly focused on the improvements brought about by the application of new technologies sponsored by NASA or the FAA. Therefore, more details such as trajectories are considered. However, more runtime will be consumed in this model. A data-driven agent-based model was also tested to predict clusters of delayed airports in the United States of America [17]. In addition to this, a queuing model, LMINET [18], was designed and used to estimate the throughput benefit of NextGen programs. Given that the model could not estimate flight delays in inclement weather, an updated model, LMINET2 [19] was developed and flight schedules were added. Focusing on the propagation of flight delays, Pyrgiotis et al. designed another queuing network model [20]. Moreover, epidemic models and machine learning technologies have been applied to address the problem of the delay propagation of flights and airports [21–24]. Specially, Li et al. presented an epidemic model for reproducing the delay propagation in airport networks with the consideration of the complex interactions between airports [23]. Liu et al. used machine learning algorithms to analyze air traffic management actions and achieve the prediction of GDP incidence [24].

However, there are some critical issues to be solved when these models are used for delay prediction in the phase of tactical Air Traffic Flow Management (ATFM). Specifically, most of these models attempt to control the degree of system refinement to achieve a tradeoff between accuracy and the time-consuming computations for predicting and analyzing delays [25], while more detailed and real-time parameter acquisition is not emphasized. For example, the minimal ground turnaround time is only a function of an aircraft seat category [19], or obtained by percentile values [26], or substituted by a constant [27]. The daily differences and carriers are ignored in these studies. It should be noted that it is the change of these time-varying parameters that causes flight delays in practice. Additionally, the computational time of up to several hours makes these simulation methods difficult to apply to the flight delay prediction in the tactical phase [20,28]. Although the epidemic model developed by Li et al and the machine learning algorithms implemented by Liu et al. can predict airport delays in the short time or in the tactical phase, both of them generated a result of aggregated delays rather than individual flight delays, in which the differences such as carries and aircraft types between flights operating in airports were simply ignored [23,24].

In order to fill these gaps, we developed an agent-based model to predict flight delay for the level of an entire air traffic network, and its critical time-varying parameters were acquired by data mining algorithms. In the model, aircraft and airports were regarded as two types of agents, and their state transition constituted the basis of the dynamics of the ATM system. The estimated elapsed time (EET) and minimum turnaround time (MTT) were two key factors that affected flight operations in the actual ATM system, and these factors were introduced into the delay prediction model. Another crucial parameter, expected departure time (EDT), was used to represent the possible waiting time with GDPs or other reasons in this study. Specific algorithms were selected to estimate these key parameters after analyzing their features. More specifically, the EET and the MTT required for flights are obtained from two Random Forest regression (RFr) models, while a conditional probability method was proposed for estimating the EDT. The difference between individual flights and the operating environment such as airport congestion can be incorporated into our parameter models compared to existing research [26,29,30]. Flights derived from the schedule are placed in such an agent-based delay prediction model to simulate their operation. Accordingly, the departure and arrival times of flights in the schedule were obtained using the state transition, and the flight delay could be calculated through the related delay criteria. It should be noted that we did not overly refine the agent-based model because the main object of this study was only to predict flight delays in the entire ATS in the tactical phase rather than identifying the flight operation in detail (such as aircraft interactions on the ramps and the taxiways). The differences between flights were considered in our model compared to the epidemic model and the machine learning algorithms mentioned before [23,24], and individual flight delays as output can help airline managers and passengers to focuse on their related flights so that they can take action to reduce their losses.

The contributions of the model are presented here are as follows. First, the model could be used to predict individual flight delays in the phase of tactical ATFM by incorporating real-time information (for example, flight planning, flight statuses, and weather conditions) into the model directly. Second, the key parameters of air traffic operation, such as the estimated elapsed time and minimum turnaround time, were explored and modeled in data mining methods. To the best of our knowledge, this study is the first to combine agent-based modeling and simulation with data mining algorithms for the purpose of the prediction of air traffic delays. Last but not least, the delay prediction model was built based on an entire airport network, and more flights were included compared to a 34 OEP airport network.

The remainder of this paper is organized as follows. Section 2 describes the framework of the delay prediction model and the state transition algorithm of agents. The critical parameter models based on data mining technologies are presented in Section 3, the factors that affect the parameters and the reasons for the selection of each parameter model are also analyzed and discussed. Section 4 describes the generation of the critical parameter model and the testing of the performance of the delay prediction model by the selection of test days with different degrees of delay. The positive effect of introducing parameter models and the negative impact of increasing the prediction horizon on the prediction performance were also studied. Section 5 provides a summary of conclusions and future work.

## Modeling overview

### Architecture of the agent-based flight delay prediction model

As typical methods for analyzing and understanding complex systems, agent-based modeling and simulation have become more and more widespread across many application domains [31]. An Air Traffic Management (ATM) system is a typical example of a complex system involving many heterogeneous individuals (e.g., flights, airports, air traffic controllers, etc.) operating in an intricate and constrained environment (air routes, time schedules, international regulations, etc.). The interaction between these individuals guided by their own local rules shows emergent behavior and nonlinear aggregation [3]. These characteristics of ATM systems bring challenges to applying analytical models to explore system dynamics. Agent-based methods (ABMs) provide convenience for modeling such systems and being able to compensate for the inability of analytical methods.

The level of abstraction of agent-based modeling is an issue that needs to be properly weighed to deal with the scale and complexity of the problem to be solved in a system. Although agent components described at a high level of abstraction could result in efficient systems, a great amount of computation time is usually required, as well as extensive input preparation [25]. Specifically, running a model for an ATM system defined at a microscopic level and the consideration of details (such as aircraft approaches, runways, taxiways, aircraft movements, bus movements, and passenger behavior during check-in) typically requires several hours [20]. Obviously, this kind of agent-based model does not meet the needs of tactical delay prediction (the future flight delay information should be given several hours in advance) due to the lack of necessary real-time information inputting.

**Fig 1:**
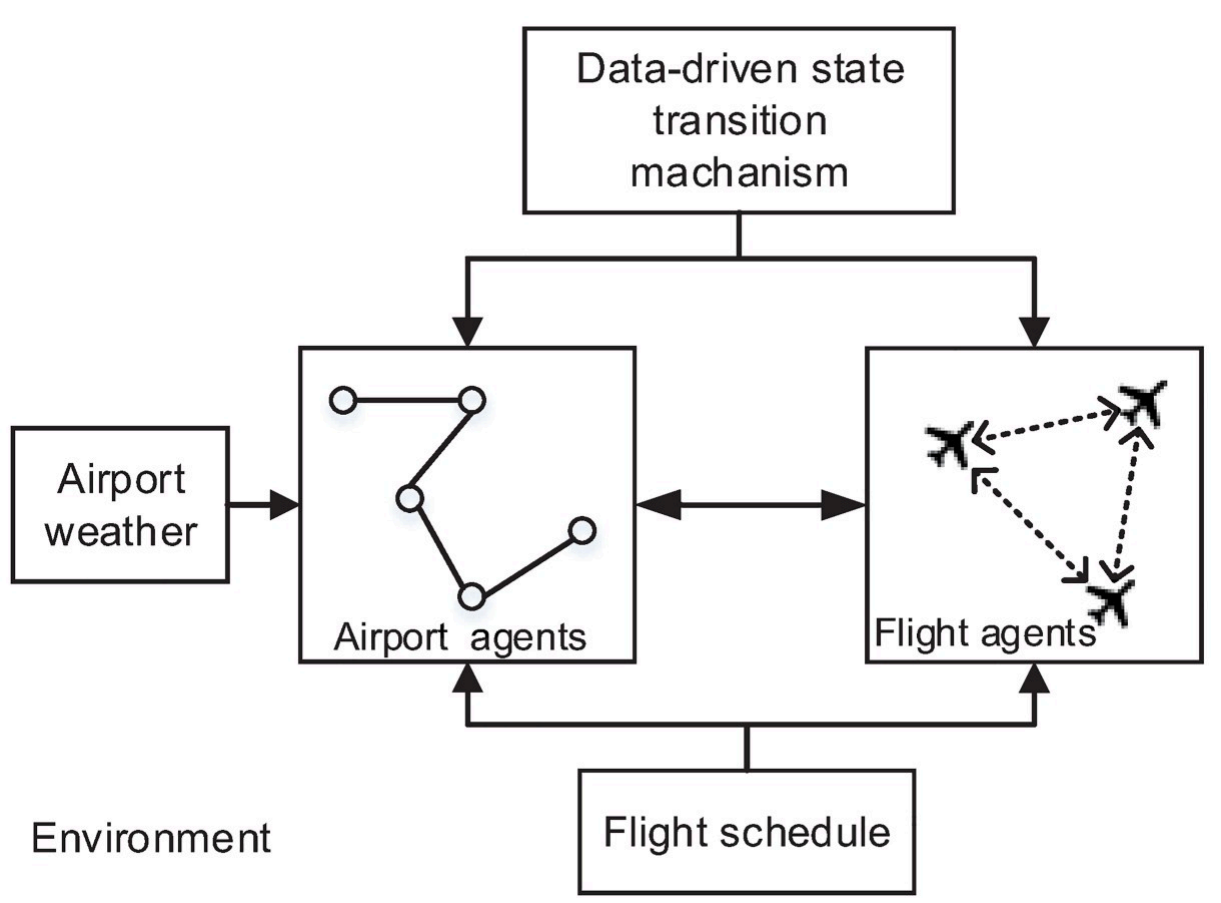
The architecture of the agent-based flight delay prediction model.

For the purpose of flight delay prediction with the entire ATM system in the tactical phase, we proposed an agent-based model with a mesoscale abstraction (Fig 1). In the model, aircraft and airports were defined as two types of agents simply but purposefully. A flight agent could:

- Acquire the flight schedule from the environment and determine its own origin and destination.
- Communicate with its origin airport agent and destination airport agent and acquire their states. The state of the departure airport agent (or the arrival airport agent) determined whether a flight agent could depart (or arrive).
- Communicate with other flight agents and acquire their states.
- Update its state according to information regarding other agents and the mechanism of flight agent state transition.

An airport agent could:

- Acquire the flight schedule from the environment and determine which flight agents interacted with it.
- Communicate with flight agents and acquire their state and the state duration.
- Perceive meteorological information from the environment and update its own capacity.
- Update its state according to its mechanism of the state transition.

Overall, the flight delay prediction model was activated by the flight schedules and meteorological information of airports. The departure times and the arrival times of flights operating in this airport network could be obtained through their state transition. Then the flight delay was computed using the difference between the simulated departures and arrival times and the scheduled departures and arrival times.

### Agent state definition

Similar to an ATM system in practice, the state transition of a flight agent was usually triggered by a series of time variables. For the sake of describing the mechanism of the agent state transition, four types of time variables needed to be clarified and defined according to the actual flight operation procedure.

- The earliest available departure time (EADT): The earliest time a flight was permitted to depart considering the fact that some flights might depart before the scheduled departure time because of adequate preparation.
- The expected departure time (EDT): The expected departure time for a flight considering GDPs and the carrier reason.
- The estimated elapsed time (EET): The requisite travel time from gate departure to gate arrival for flights.
- The minimum turnaround time (MTT): The minimum time of accomplishing provision for operations like passenger disembarkation, cabin cleaning, refueling, a pre-flight inspection of the aircraft, passenger boarding, luggage and cargo loading, and programming of the Flight Management System.

According to the time interval that they fell into, the states of the flight agents were divided into six categories. The flight agents migrated between these states according to the operating conditions. The implications of the states were as follows:

- 0-Unallocated: The initial state of the flight agents.
- 1-Turnaround: The state of the flight agents when they were accomplishing provisioning for operations like passenger disembarkation, cabin cleaning, refueling, a pre-flight inspection of the aircraft, passenger boarding, luggage and cargo loading, etc.
- 2-Prepared: Flight agents had completed ground service and were about to start their itineraries, but they had not yet been permitted to depart due to not reaching the expected time for departure and the earliest time available for departure.
- 3-Waiting: Flight agents that had gone through the “prepared” state but had not been permitted to depart because of the capacity restrictions of their departure airports.
- 4-Flying: Flight agents were in the process of travelling from gate departure to gate arrival.
- 5-Arrived: Flight agents had arrived at the destination during the course of the day.

Accordingly, two categories of airport agents were defined according to whether the airport had the ability to meet the departure or arrival requests for a flight in a time interval after providing services for other flights (the first-come-first-served principle was usually adopted). Specifically, the affirmative situation was called an unsaturated state; otherwise, the state was called a saturated state.

### Data-driven mechanism of flight agent state transition

The state transitions of the flight agents constituted the dynamic foundation of the flight delay prediction model. Similar to the real-life flight operation, their state transitions were controlled by some key parameters. It should be noted that the difference of some terminologies between the flight agents and the flights, airport agents, and airport is not overemphasized for the rest of the paper.

The notations used in the model are defined in Table 1. We let $x_{i}^{t}(s)$ denote the state of the flight agent *i* at time *t* while $x_{i}^{t-1}=s$, *s*∈{0, 1,…,5}. The state transitions of a flight could be formulated as shown below.

$$x_{i}^{t}(0)=\left\{ \begin{array}{l} 1,\;if\;x_{i^{-}}^{t}=5\;or\;i\notin IF\\ 0,\;otherwise \end{array}\right.$$ (1)

$$x_{i}^{t}(1)=\left\{ \begin{array}{l} 2,\;if\;TT_{i}^{t}\geq MTT_{i}\\ 1,\;otherwise \end{array}\right.$$ (2)

$$x_{i}^{t}(2)=\left\{ \begin{array}{l} 3,\;if\;t\geq EDT_{i}\\ 2,\;otherwise \end{array}\right.$$ (3)

$$x_{i}^{t}(3)=\left\{ \begin{array}{l} 4,\;if\;t>STD_{i}-THR\;and\;y_{ad_{i}}^{t}=0\\ 3,\;otherwise \end{array}\right.$$ (4)

$$x_{i}^{t}(4)=\left\{ \begin{array}{l} 5,\;if\;ET_{i}^{t}>EET_{i}\;and\;y_{aa_{i}}^{t}=0\\ 4,\;otherwise \end{array}\right.$$ (5)

$$x_{i}^{t}(5)=5$$ (6)

**Table 1:** Notations and descriptions.

Notation	Description
*A*, *a*	The set of airport agents in the air traffic network, *a*∈*A*, representing the airport agent *a*.
*t*, *t*_0_, Δ*t*	The time *t*_0_ is the lower bound of a time interval Δ*t*, and [*t*,*t*_0_]⊂Δ*t*.
$C_{a}^{\Delta t}$	The capacity of the airport agent *a* in the period Δ*t*.
$T_{a}^{t}$	The throughput of the airport agent *a* in [*t*_0_,*t*].
*F*	The set of flight agents in the air traffic network.
*IF*	The set of initial flight agents in the course of the day.
*SF*	The set of flight agent states.
*SA*	The set of airport agent states.
$y_{a}^{t}$	The state of the airport agent *a* at *t*. $y_{a}^{t}\in \left\{0,1\right\}$, 0 indicates that the state of an airport was *non-saturated*, 1 indicates that the state of an airport was *saturated*.
*THR*	The threshold for early departure.
$x_{i}^{t}$	The state of the flight agent *i* at, $x_{i}^{t}\in \left\{0,1,2,3,4,5\right\}$
*i*^−^	*i*^−^ is the upstream flight agent of the flight agent *i*.
*ad*_*i*_, *aa*_*i*_	The origin airport and the destination airport agent of the flight agent *i*.
*STD*_*i*_	The scheduled departure time of the flight agent *i*.
*EET*_*i*_, *MIT*_*i*_	The estimated elapsed time and the minim turnaround time of the flight agent *i*.
*EDT*_*i*_	The expected departure time of the flight agent *i*.
$ET_{i}^{t}$, $TT_{i}^{t}$	The elapsed time and the turnaround time of the flight agent *i* at *t*.
*DT*_*i*_, *AT*_*i*_	The departure time and the arrival time of the flight agent *i*.

The first item in Eqs (1)–(5), i.e., $x_{i}^{t}(s)=s+1$, *s* = 1,2,3,4,5, indicates the state transitions of the flight agent at the time *t*. Specifically, Eq (1) denotes that the state of the agent *i* would update from “0-unallocated” to “1-turnaround” at *t* if its upstream flight agent had been under “5-arrived”. The agent *i* on “1-turnaround” would update to“2-prepared” in Eq (2) if it had experienced enough time for its turnaround at *t*. Eq (3) denotes that the agent *i* on “1-turnaround” would update to “2-prepared” at *t* for the case that it had experienced enough time due to the carrier and the GDP reason. Eq (4) denotes that the agent *i* on “3-waiting” would update to “4-flying” without the capacity restrictions of the origin airport agent at *t*. Eq (5) denotes that the agent *i* on “4-flying” would update to “5-arrived” if it had experienced block time being airborne and on the ground at *t*. In contrast, the second item of the above Eqs (1)–(5) indicates that the state of the flight agent *i* failed to change at *t* since the conditions were not met. In particular, Eq 6 means that the state was not changed after the flight arrived.

The more understandable mechanism with the state transitions of flight agents is explained in Algorithm 1. The interaction between flight agents was achieved in the process of looking for the state of their upstream flight agents. With the help of these rules, some significant results influenced by several key parameters (such as the expected departure time, the minimum turnaround time, and the estimated elapsed time) including the departure time and arrival time of the flight agents could be obtained. These parameters were estimated through the data mining methods discussed in next Section 3.

**Algorithm 1**: The state transitions of the flight agents from *t-*1 to *t*

**Input**: $x_{i}^{t-1}$, $ET_{i}^{t-1}$, $TT_{i}^{t-1}$, $C_{ad_{i}}^{\Delta t}$, $C_{aa_{i}}^{\Delta t}$, *EET*_*i*_, *MIT*_*i*_, *STD*_*i*_, $T_{ad_{i}}^{t}$, $T_{aa_{i}}^{t}$, *THR*

**Output**: $x_{i}^{t}$, $ET_{i}^{t}$, $TT_{i}^{t}$, $T_{ad_{i}}^{t}$, $T_{aa_{i}}^{t}$, *DT*_*i*_, *AT*_*i*_

1. **Begin**

2. i**f** $x_{i}^{t-1}=0$

3. look for the state of the agent *i*^−^, $x_{i^{-}}^{t-1}$, at *t* according to its tail number

4. i**f** $x_{i^{-}}^{t-1}=5$

5. $x_{i}^{t}\leftarrow 1$, $TT_{i^{-}}^{t}\leftarrow 0$

6. **if** $x_{i}^{t-1}=1$

7. **if** $TT_{i}^{t}>=MTT_{i}$ 7. $x_{i}^{t}\leftarrow 2$

8. **else** $TT_{i}^{t}\leftarrow TT_{i}^{t-1}+1$

9. i**f** $x_{i}^{t-1}=2$

10. Compute *EDT*_*i*_

11. **if** *t*≥*EDT*_*i*_

12. $x_{i}^{t}\leftarrow 3$

13. **if** $x_{i}^{t-1}=3$ and $T_{a_{i}^{d}}^{t}<C_{a_{i}^{d}}^{\Delta t}$

14. $x_{i}^{t}\leftarrow 4$, *DT*_*i*_←*t*, $T_{ad_{i}}^{t}\leftarrow T_{ad_{i}}^{t}+1$, $ET_{i}^{t}\leftarrow 0$

15. **if** $x_{i}^{t-1}=4$

16. **if** $ET_{i}^{t}\geq RET_{i}$ and $T_{aai}^{t}<C_{a_{i}^{a}}^{\Delta t}$

17. $x_{i}^{t}\leftarrow 5$, *AT*_*i*_←*t*, $T_{aa_{i}}^{t}\leftarrow T_{aa_{i}}^{t}+1$

18 **else** $ET_{i}^{t}\leftarrow ET_{i}^{t-1}+1$

19. **End**

### Data-driven mechanism of airport agent state transition

The state of the airport agents was determined by the airport capacity and the number of aircraft that the airport agents served at the time interval [*t*_0_,*t*]. We let *k* indicate the number of flights, including departure and arrival at the airport a. As shown in Eq (7), the state of airport agent *a* was set to 0 (indicating the unsaturated state) when its throughput was less than the capacity during [*t*_0_,*t*]. This meant that the airport a could provide services with the arrival and departure at *t*. Accordingly, the state of airport agent *a* would be set to 1 if its throughput was less than the capacity during [*t*_0_,*t*]. The throughput of the airport agent *a* could be calculated as shown in Eq (8).

$$y_{a}^{t}=\left\{ \begin{array}{l} 0,\;T_{a}^{t}<C_{a}^{\Delta t}\\ 1,\;otherwise \end{array}\right.$$ (7)

$$T_{a}^{t}=\left\{ \begin{array}{l} T_{a}^{t-1}+\sum_{i_{a}=1}^{k}c_{i_{a}}^{t},\;t>t_{0}\\ \sum_{i_{a}=1}^{k}c_{i_{a}}^{t},\;t=t_{0} \end{array}\right.$$ (8)

$$c_{i_{a}}^{t}=\left\{ \begin{array}{l} 1,\;if\;x_{i_{a}}^{t}=3\;or\;5,\;and\;x_{i_{a}}^{t}\neq x_{i_{a}}^{t-1}\\ 0,\;otherwise \end{array}\right.,\;i_{a}=1,2,\ldots ,k$$ (9)

where $c_{i_{a}}^{t}$ indicates whether the flight *i*_*a*_ at the airport *a* had departed or arrived at *t*. Therefore, $T_{a}^{t}$ was *a* cumulative process based on whether the aircraft had been served, which could be obtained based on Eq (9). In Eq (9), $c_{i_{a}}^{t}=1$ meant that the flight agent had undergone *a* state transition and changed to 4 or 5 from *t*−1 to *t*. Its practical significance indicated that the flight *i*_*a*_ had completed a departure or arrival at the airport *a* at *t*. At that time, the throughput of the airport *a* was increased by 1. The airport capacity (AC), another key variable that gave rise to the transition of airport agents, is also discussed in Section 3.

The overall process of the flight delay prediction model is shown in Algorithm 2. In the input part of the model, parameter models (PMs) obtained with data mining methods were used to generate the key parameters, including the expected departure time (EDT), the estimated elapsed time (EET), and the minimum turnaround time (MTT), which controlled the state transition of two types of agents. The state of all the flight agents was set as 0-unallocated in the initialization. As a result, the departure and arrival times of flights in the schedule could be obtained through the operation of the model. Then the delay of these flights could be calculated.

**Algorithm 2**: The delay prediction process of the agent-based model.

**Input:** The flight schedule, meteorological information, PMs, the start time *t*_*s*_, and the end time *t*_*d*_.

**Output:** The predicted departure time and the arrival time *DT*_*i*_, *AT*_*i*_ for *i*∈*F*

1. **initialize** *SF* = 0, *t* = *t*_*s*_

2. **while** *t*≤*t*_*d*_

3. **for** each flight *i* in *F*

4. **if** $x_{i}^{t}\neq 5$

5. execute **Algorithm 1**

6. update *SF* and *SA*

7. **end for**

**8.** *t*←*t*+1

9. update *SF*, *SA*, $T_{ad_{i}}^{t}$ and $T_{aa_{i}}^{t}$

10. **end while**

## Critical parameter modeling

As mentioned earlier, several parameters, the expected departure time (EDT), the minimum turnaround time (MTT), the estimated elapsed time (EET), and the airport capacity (AC), played an important role in the state transitions of the flight agents and airport agents. In practice, the on-time performance of the flights in an ATM system is highly related to these parameters. For example, the decline of airport capacity due to severe weather caused a large proportion of flight delays in the ATM systems [32]. Hence, the accuracy of parameter estimation would largely influence the performance of the agent-based prediction model. As described in this section, we proposed several parameter models to estimate the above parameters to underpin tactical flight delay prediction. The parameter models (PMs) were trained and constructed using historical data from 2017 in the United States of America.

### Data source

The data we used to train the models could be divided into two categories, namely the flight data and the meteorology data for airports. The flight data was acquired from the On-Time Performance Database of the Bureau of Transportation Statistics (BTS) database, while the meteorological data for the airports was derived from METAR.

**Fig 2:**
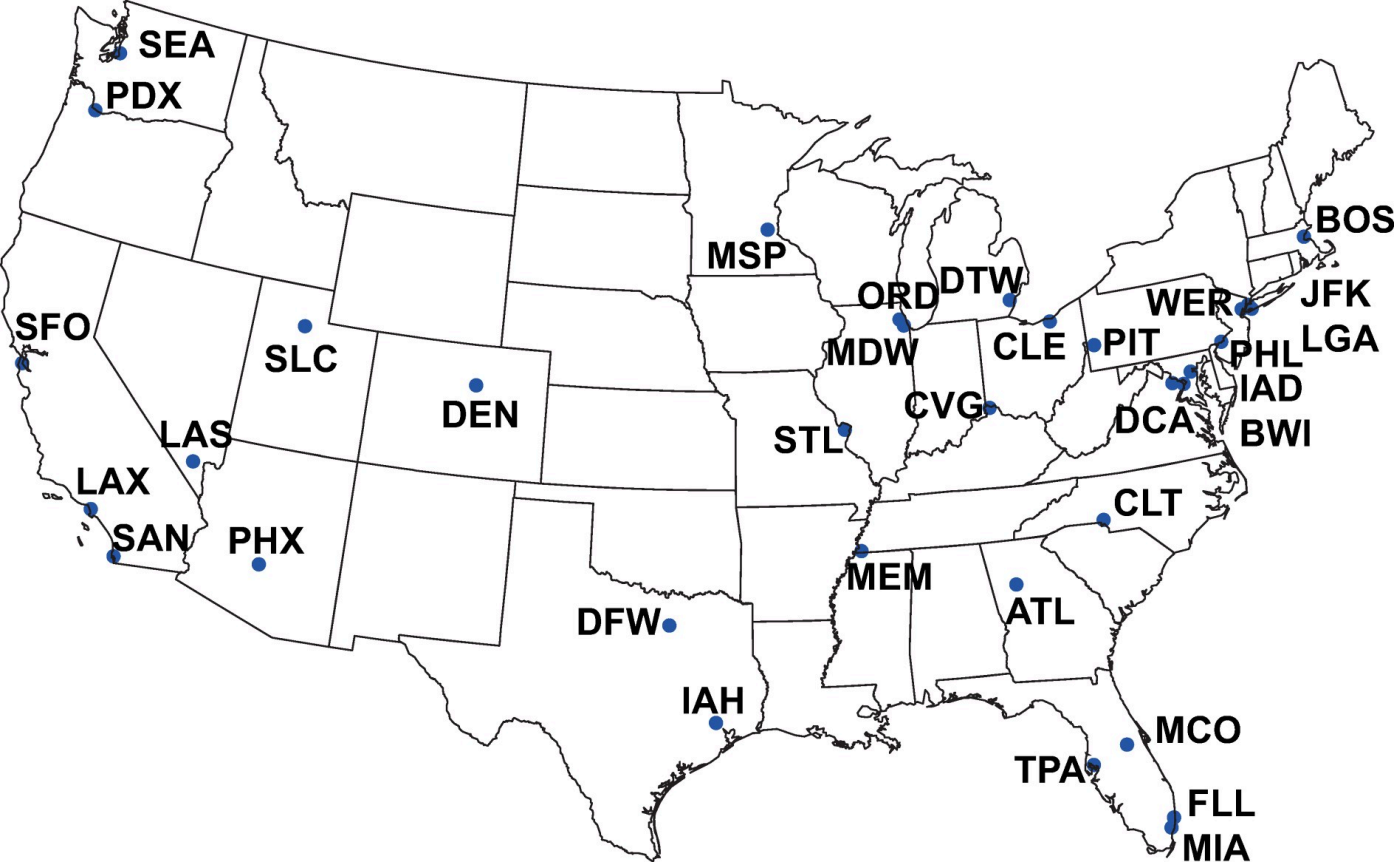
The busiest 34 airports in the United States of America. The map was created by Python 3.6 with the help of free vector and raster map data from Natural Earth (http://www.naturalearthdata.com/) and the location data of airports from BTS (https://www.bts.gov/).

In contrast to the simplified National Airspace System (NAS) that consists of 34 OEP airports (Fig 2) [20], a network with more airports was built in our study. A large number of the less busy airports, however, had fewer flights than these 34 busiest airports, but flights between these less busy airports and the 34 airports still took up the service time of the latter, which affected the other flights operating at the 34 airports because of the interactions in the airport networks. Furthermore, the propagation of delays was caused by this exact kind of interaction with agents. Therefore, instead of using the simplified network, we took account of all the flights and airports in the On-Time Performance Database.

One year of flight data during 2017 was obtained and used to train the parameter models. These data included all the necessary information for training the parameter models (PMs), such as the flight number, tail number, departure airport, arrival time, scheduled departure time, and scheduled arrival time. In addition, aircraft itineraries and airport demands were also obtained from the On-Time Performance Database.

### Expected departure time model

The expected departure time (EDT) was a prerequisite for determining the departure of the flight strictly, and it could be regarded as the initial departure qualification in our model. The actual departure times of flights might have deviated from the scheduled times due to the implementation of GDPs or the reasons of the airlines. The inaccessibility and uncertainty of these factors made it difficult to predict the expected departure time of the flights accurately.

Alternatively, a conditional probability model was presented for predicting the expected departure time in this study. The expected departure time of a flight agent was determined by its current delay in the model. However, the possibility of obtaining the initial departure qualification (i.e., the condition of updating the flight agent state from 2 to 3) for a flight only needed to be explained by the delay time of the flight at *t*. This was logical from a statistical point of view. Since the delays for flights at *t* were different, the probability during [*t*,*t*+Δ] was different. For that case, the delay probability of a flight depended on the delay probability distribution as well as its delay time. This statistical approach was able to integrate with the delayed time due to GDPs and carrier reasons, and more real-time information could also be incorporated.

We defined *F*(*x*) as the probability distribution function of the departure delay, and the delay time of the flight agent *i* at *t* was $t_{i}^{delay}=x$. Then the possibility of obtaining the initial departure qualification for this flight at *t*+Δ could be expressed as (10).

$$P_{t+\Delta }^{i}=P\left(d=1|t_{i}^{delay}=x\right)=\frac{P\left(x<t_{i}^{delay}\leq x+\Delta \right)}{P\left(t_{i}^{delay}\geq x\right)}=\frac{F(x+\Delta )-F(x)}{1-F(x)}$$ (10)

where *d* = 1 meant that the initial departure qualification for the flight *i* was available, and *d* = 0 meant that it was unavailable.

To produce a specific number for the expected departure time, we presented a random number method. Specifically, a random number *r* was introduced that followed a uniform distribution in the interval (0,1). It should be noted that the method of random numbers might bring slight uncertainty to the model, but it was not unreasonable because the number of flights was considerably large and the randomness could be offset to some extent. Furthermore, the randomness was merely relative. There was randomness on an individual basis, but these results still obeyed the fitted distribution from an overall perspective. Moreover, the result of this model was just a parameter in the agent-based delay prediction model rather than the final departure time.

We could obtain the expected departure time for the flight *i* using Eq (11) when $r<P_{t+\Delta }^{i}$. The probability distribution function could be obtained with Eq (12).

$$EDT_{i}=\min\left[t,EADT\right]$$ (11)

$$F(x)=\int_{-\infty }^{x}f(t)dt$$ (12)

where *f*(*t*) is the probability density function of *F*(*x*).

### Estimated elapsed time and minimum turnaround time

The estimated elapsed time and minimum turnaround time are also crucial parameters in controlling flight agent state transitions. Airlines often add buffers to the elapsed time and turnaround time to reduce delay propagation [26,33,34]. As a result, the scheduled time is usually slightly longer than the normal or minimum time. Therefore, not only will the flight not be delayed, but it may also depart and arrive earlier than the schedule when operating in favorable conditions, such as good meteorological conditions.

There were some notable limitations for the elapsed time and turnaround time of flights in the current research. A percentile method is usually adopted for obtaining the minimum elapsed time and turnaround time [26,30]. This method enables researchers to acquire the time for eliminating the buffer padded by airlines to some extent. Nonetheless, the difference between flights in operation is usually ignored with this method. For example, the additional elapsed time may be needed for the cases of the congestion of airspace or airport surfaces. Additionally, the magnitude of buffers may vary from the airline operating strategy. Another method to generate the estimated elapsed time usually relies heavily on aircraft performance models, along with either parametric or physics-based trajectory models [29]. They usually begin by estimating the flight trajectory, which includes the lateral flight path together with altitude and speed profiles, and then calculate the time required to fulfill the predicted trajectory. These models by themselves fail to account for external operational circumstances which can directly affect the actual flight profile, such as weather phenomena or airport congestion. The same is true to the minimum turnaround time.

To this end, we developed a model to perform data-based predictions for the estimated elapsed time and minimum turnaround time of flights. The two random forest regression (RFr) models were trained for the estimation of the above two critical parameters in this research. Random forest is an ensemble learning method for classification and regression that operates by constructing a multitude of decision trees at training time. It provides an improvement over bagged trees by way of a small tweak that decorrelates the trees. RF worked well for a large number of variables [35] and was verified to be effective in the predicting response variables in an ATM system [36].

There was a problem with getting the training data for the minimum turnaround time since it was usually very hard to get the finish time of the ground service, which was a key point for calculating the turnaround time of flights. To solve this problem and to obtain the data that enabled the minimum turnaround time to be represented more veritably, we only considered the flights that had been delayed (more than 30 min) in the upstream segment and that had departed normally. For that case, the turnaround time after absorbing the partial delay could represent the minimum turnaround time [30].

The possible factors affecting the estimated elapsed time and minimum turnaround time were introduced in these two models. In particular, *the scheduled elapsed time*, *aircraft type (the number of seats)*, *carriers*, *OD pairs*, *weeks*, and *seasons (the day of the year)* were considered as explanatory variables for the estimated elapsed time model (EET model). Among these factors, we used the number of seats to represent the aircraft type, and we used the day of the year to represent the season. In particular, the carriers denoted their operating strategy, and the OD pair was defined by its demands. In the minimum turnaround time model (MTT model), the factors including *the schedule turnaround time*, *the scheduled elapsed time*, *the scheduled elapsed time of the upstream flight*, *the aircraft type (the number of seats)*, *seasons (the day of the year)*, *week*, and *airports* were considered. The scheduled elapsed time for the flight and the upstream flight represented the distance traveled by the aircraft. In general, the longer the flight distance, the longer the turnaround time required to be. In addition, airports were represented by their demands. In both models, the temporal variables (the season and the day of the week) included time-varying trend of the MTT and the EET in a long time scale; the type of aircraft implied aircraft the differences of physical attributes such aircraft performance; the airports or OD pairs denoted the level of airport congestion.

### Airport capacity setting

The airport capacity is usually defined as the hourly throughput that an airport’s runways are able to sustain during periods of high demand [37]. It is easily affected by weather conditions. Weather in the terminal area could be divided into visual meteorological conditions, instrument meteorological conditions, and marginal meteorological conditions, along with the corresponding capacity types called the visual capacity, instrument capacity, and marginal capacity, respectively. Airport capacity generally decreased during inclement weather conditions, which might include poor ceilings and visibility (requiring different air traffic control procedures), unfavorable winds (so the best runway configuration could not be used), or heavy precipitation. The extent of the reduction in the capacity rates during operations in instrument conditions (as compared to visual conditions) varied widely across the 30 airports. The FAA evaluated the capacity of more than 30 core airports under these three conditions [37], which was typically used to model delay propagation [20]. The different runway configurations and operational procedures in adverse weather were considered in the airport capacity files.

The capacity of core airports in this study was set based on the capacity files and weather conditions. First, the meteorological condition of an airport in a time interval was determined according to METAR, which provided necessary weather characteristics such as the visibility (VIS) and ceiling (CEI). Then the airport capacity in the time interval corresponded to its meteorological condition. Additionally, the capacity of the non-core airports was simply represented by its demands.

## Results

### Generation of critical parameter models

**Fig 3:**
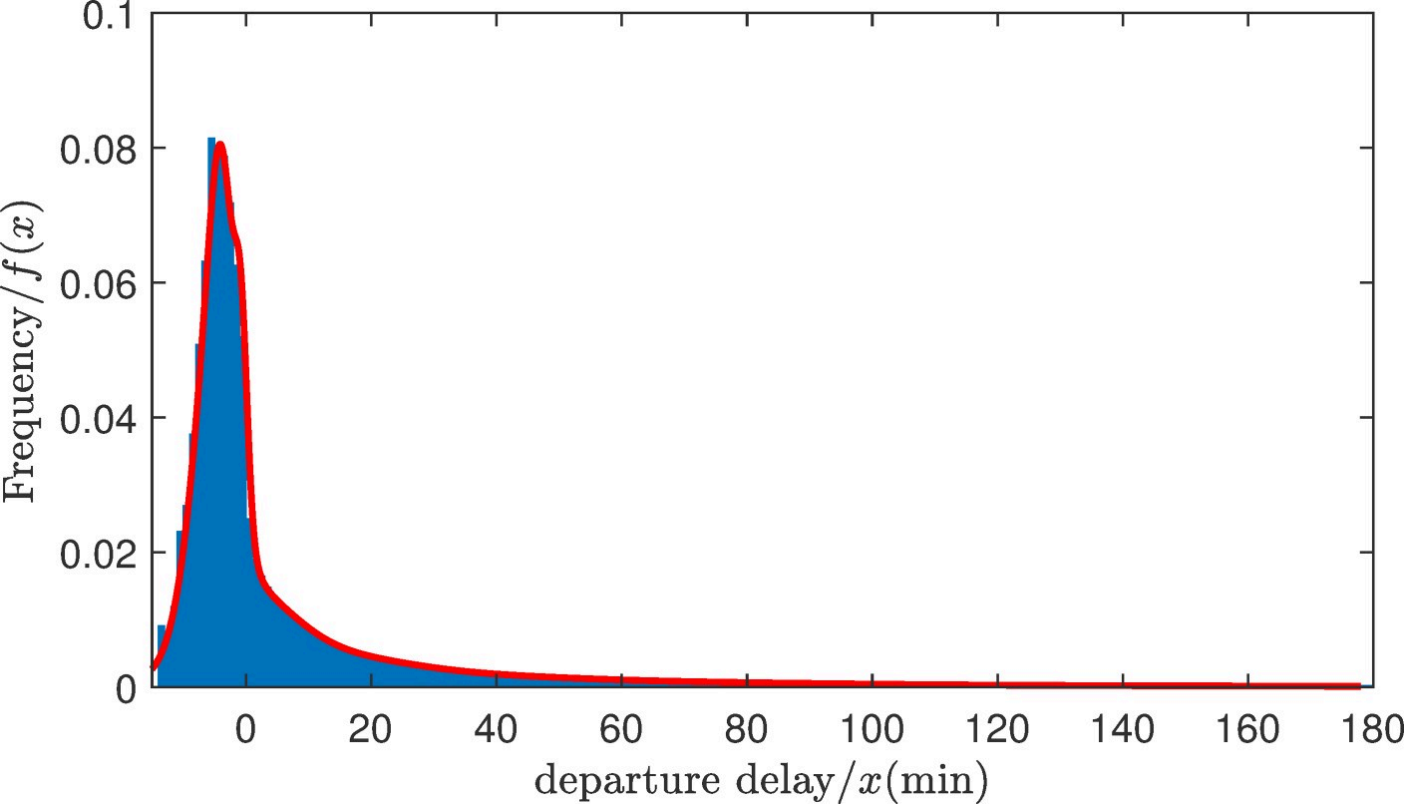
Histogram of the frequency distribution of the departure delay and the probability density function of the departure delays.

**Fig 4:**
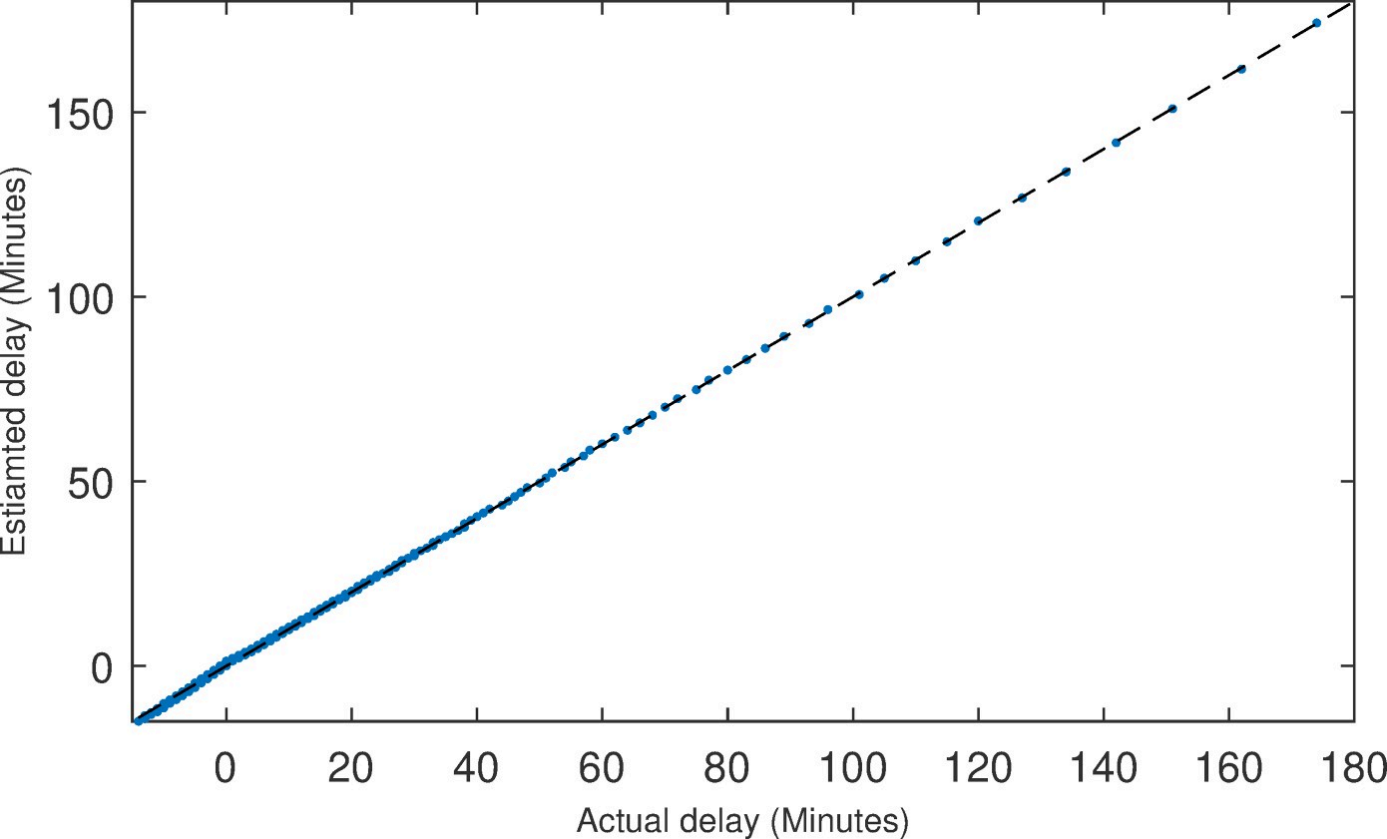
The Q-Q plot.

The flight data during 2017 were used to construct the expected departure time model. After deleting the cancelled and diverted flights, which accounted for about 1.7% of the total amount, we obtained 5,579,410 records in total for the departure delays. A long-tailed distribution (see Fig 3) was observed by means of the histogram of the frequency distribution of the departure delays. Hence, we tried to use a Gaussian mixture distribution to fit the histogram. As a result, the probability density function of the initial departure qualification with thirty mixture components was obtained (see Fig 3). It can be seen from Fig 4 that the probability density function *f*(*t*) could be fitted effectively.

Since the expected departure time was a hidden quantity in the model and system, it was very difficult to compare the error with the real data directly. However, our purpose in introducing it was mainly to fill the gap of directly taking the planned departure time (or multiplied by a factor) as the expected departure time in existing research [20,27,28]. Therefore, we did not immediately test the performance of the expected departure time model but rather proved its effectiveness by measuring the flight delay prediction accuracy.

After filtering and integrating the flight data set from January to December of 2017, 97,421 records were used for the MTT model. Following the data using in the minimum turnaround time model, the EET model incorporated 5,579,410 records for the elapsed time of flights. We used ten-fold cross-validation to generate the training and test data sets and then tested the performance of the two models we presented.

**Fig 5:**
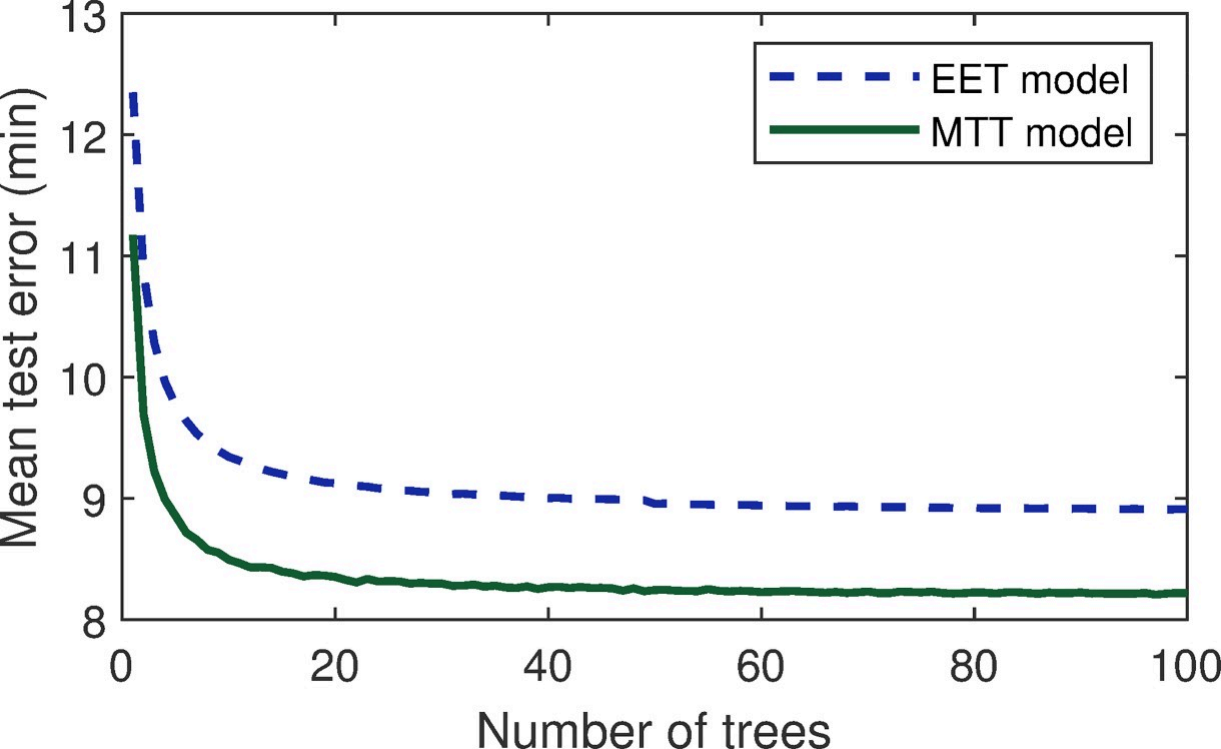
Random Forest regression (RFr) method performance with the estimated elapsed time (EET) and minimum turnaround time (MTT) models for a different number of trees.

For these two RFr models, we selected the minimum number of observations per tree leaf to be two, and the size of the random subset of variables searched at each split was chosen to be the square root of the total number of variables. Concerning the number of trees, we could see from Fig 5 that for more than 50 trees, the error curves were almost flat, and the extra trees did not improve the prediction model performance. However, it was interesting to have more than 50 trees in our models to obtain a more robust estimate of the importance of the different variables.

The ten-fold cross-validation test errors of the estimated elapsed time (EET) and minimum turnaround time (MTT) models were 8.9 and 8.2 min, respectively. These values were all lower than 15 min, which was a delay threshold value defined by the FAA. Hence, we chose the two trained models for generating the estimated elapsed time and minimum turnaround time of the flight agents in the agent-based delay prediction model. It should be noted that the estimated elapsed time and minimum turnaround time were not the final elapsed time or the turnaround time of the flights after being executed, but rather an input to the simulation. As can be seen in the rest of this paper, these two models contributed to the delay prediction model.

### Test days

In order to evaluate the performance of the delay prediction model comprehensively, thirty test days with different delay levels (low, high, and medium) were randomly selected after clustering the daily delay in 2018. The daily delay represented by the flight delay rates (the ratio between the number of delayed flights and the number of all flights) of the NAS in a given day was clustered by k-means. Table 2 summarizes the delay information of the test days. H, M, and L represent high, medium, and low delays, respectively. The values of the average departure delays were in minutes, and the flights scheduled to depart between 08:00 and 23:59 (local time) were counted. Although these test days were extracted randomly, we could also see that most of the high and medium delays occurred in summer, especially in July.

**Table 2:** Flight information and delay levels on test days.

Date	Rate of delays	Avg. departure delay [min]	Level of delay	Number of flights	Number of airports
Jul. 15	26.5%	22.1	H	20,123	334
Jun. 28	30.9%	31.3	H	20,831	330
Dec. 29	26.5%	15.7	H	18,673	331
Aug. 21	24.7%	15.3	H	19,803	330
Jul. 31	26.9%	21.9	H	20,793	332
Feb. 20	26.0%	20.7	H	18,223	317
Jul. 23	32.3%	30.8	H	20,241	336
Nov. 26	31.3%	30.8	H	19,777	329
May 16	26.4%	20.7	H	19,421	322
Jul. 21	24.6%	15.7	H	18,092	336
May 18	22.0%	13.7	M	20,447	329
Jul. 27	23.3%	22.7	M	20,252	336
Mar. 1	21.8%	15	M	19,201	318
Jul. 28	20.1%	13.8	M	18,009	336
Jul. 12	20.9%	15.1	M	20,981	334
Aug. 18	19.1%	13.6	M	16,966	334
Sept. 11	16.2%	7.1	M	19,506	327
Mar. 21	16.7%	12.7	M	15,965	322
Sept. 28	19.1%	8.6	M	18,878	326
Oct. 5	17.6%	9	M	20,739	331
Oct. 23	13.4%	5.7	L	19,939	322
Nov. 24	15.5%	7.9	L	19,973	331
Apr. 29	10.7%	4.3	L	19,612	322
Mar. 10	13.8%	5.3	L	16,898	323
Apr. 28	9.8%	4.3	L	16,200	324
Aug. 23	14.1%	7.2	L	20,870	331
Sept. 4	11.8%	5.3	L	19,321	326
Jun. 5	12.9%	5.4	L	20,056	326
Dec. 16	15.8%	8.9	L	18,460	331

Eastern Time was chosen as the benchmark time, and it was first tested from 8:00, 10:00… 22:00 to the next 2 h. Additionally, the threshold for early departure, a constant parameter in the agent-based model, was set as 10 min according to the earliest possible departure time of flights. The number of two types of agents was derived from the number of flights and airports (Table 2), and the flight schedule in the NAS guided the behavior of agents in the delay prediction model. The necessary flight and airport information, which included the tail number, scheduled departure time, origin, destination, scheduled arrival time, meteorological condition, and demands on test days, was used as input for the parameters models (PMs) and the delay prediction model.

### Performance of the delay prediction model

**Fig 6:**
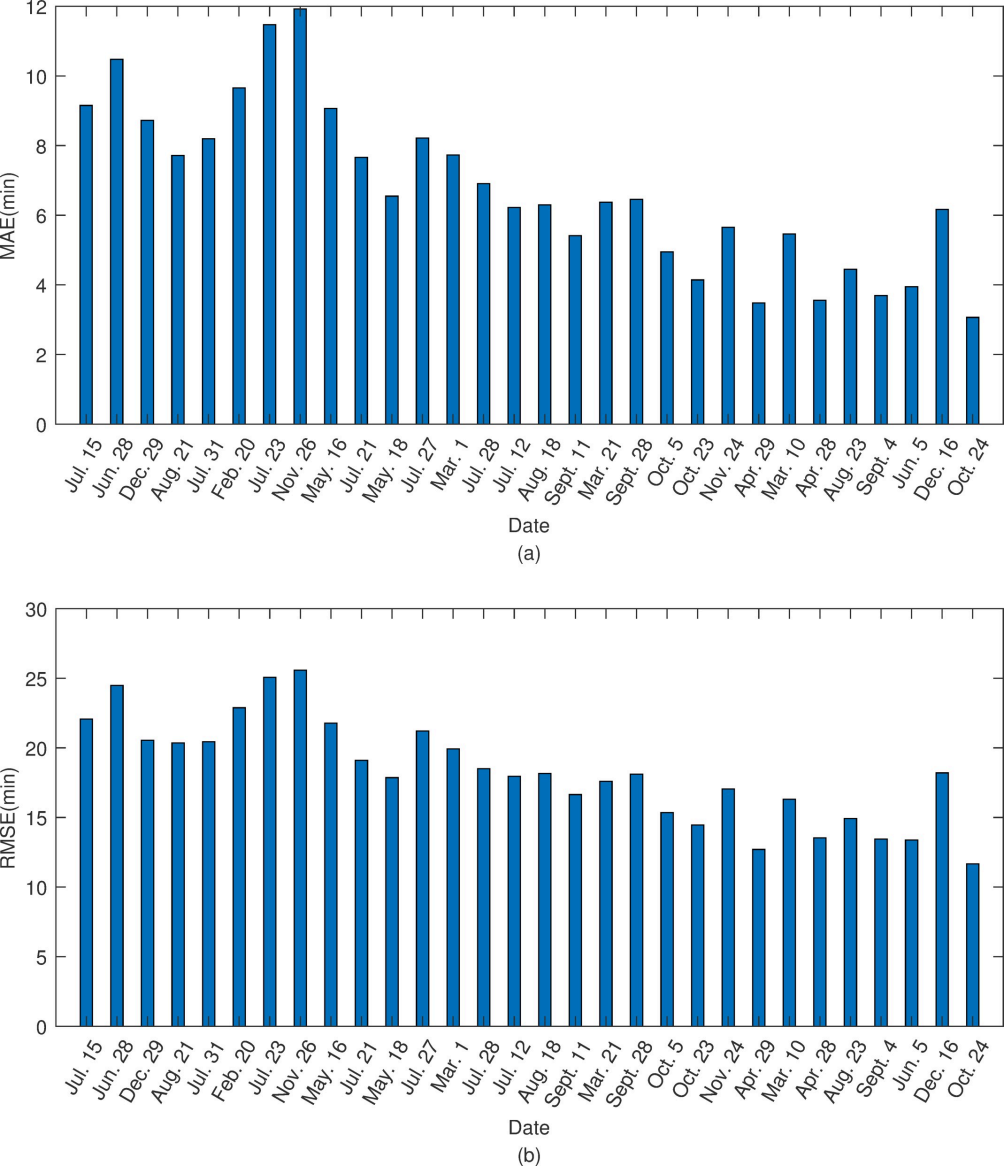
The error with test days. (a) indicates MAE of the predicted delays of thirty test days and (b) indicates RMSE of the predicted delays of thirty test days.

The mean absolute error (MAE) and root mean square error (RMSE) of the departure delays were used to measure the performance of the model. Fig 6 shows the histogram of the MAE and RMSE of the predicted delays for the thirty test days. The mean absolute error values ranged from 3.1 min (Oct. 24, low delay day) to 12 min (Nov. 26, high delay day), and the root-mean-square error values ranged from 11.7 min (Oct. 24, low delay day) to 25.6 min (Nov. 26, high delay day). Additionally, we found that the performance of the agent-based delay prediction model was closely related to the level of delay on the test day. The model performed particularly well on test days with low delays, and the MAE and RMSE were 4.4 min and 14.6 min, respectively (Table 3), while the performance decreased on test days with high delays, and the MAE and RMSE were 9.4 min and 22.2 min, respectively. Accordingly, the performance of the medium delay test days lay in between. Although the model showed slight differences on different delay level test days, the overall MAE and RMSE on the thirty test days could remain within 6.8 min and 18.3 min. Moreover, the accuracy of the classification was 89.5% with the 15 min threshold, which usually serves as a delay criterion for the FAA.

**Table 3:** The performance for three types of test days and all test days with different measurements.

Test days	MAE [min]	RMSE [min]	Accuracy
High delay days	9.4	22.2	87.1%
Medium delay days	6.5	18.1	89.3%
Low delay days	4.4	14.6	92%
All test days	6.8	18.3	89.5%

**Fig 7:**
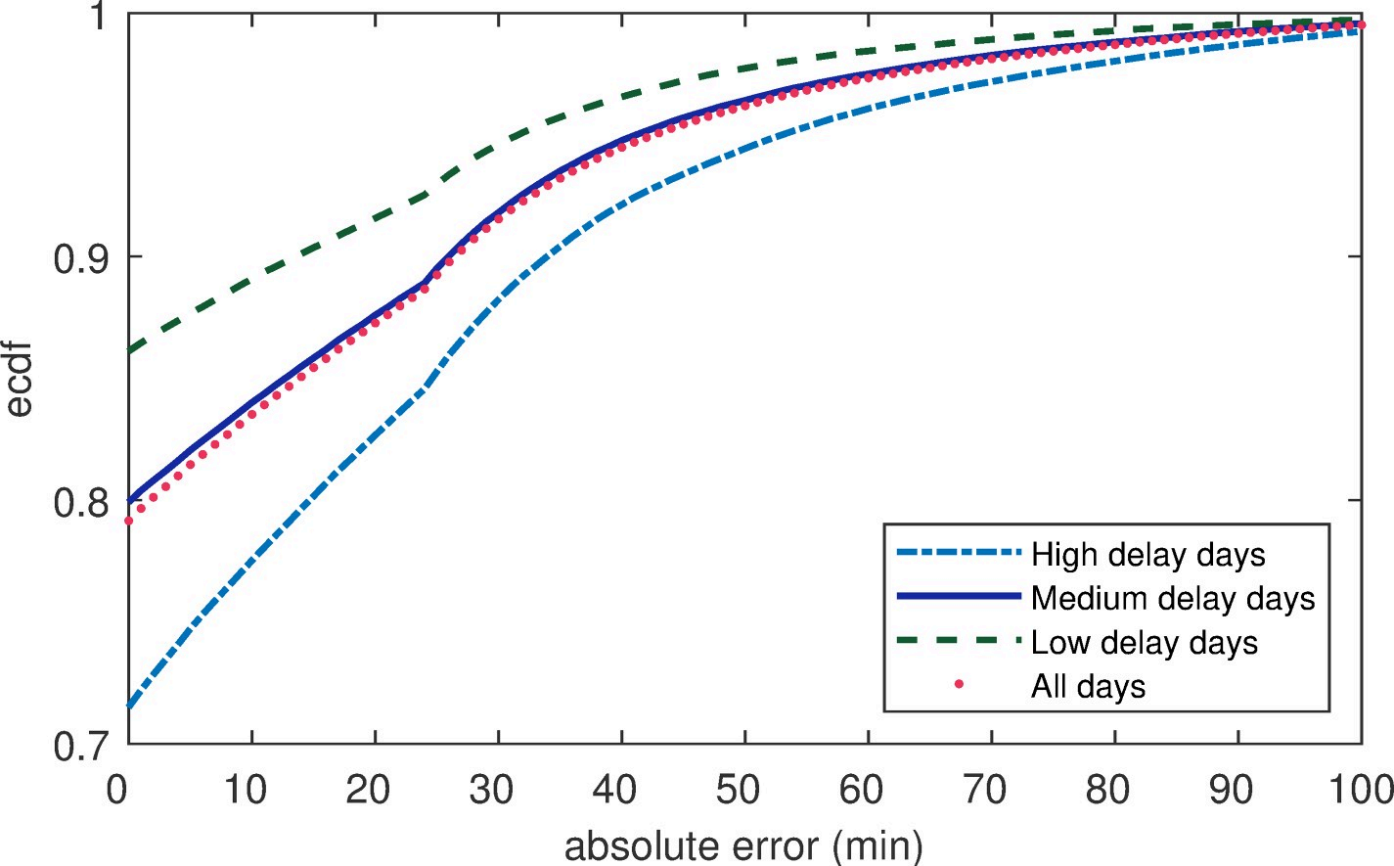
The empirical cumulative distribution function (ecdf) of the absolute errors of flight delays.

Furthermore, the empirical cumulative distributions function (ecdf) of the errors with flights on different test days was investigated. As can be seen in Fig 7, the test error values of flight delays were low, with the 90th percentile of the error distribution being 30 min. On the high-delay days, nearly 79% of the forecast errors were less than 15 minutes in our model for 2-hour forecast windows. However, no matter which types of test days were used, there were still some flights that were not correctly predicted. However, the magnitude with high error on the high delay days was higher than those for the other two types of test days. For example, Fig 7 shows that nearly 5% of flight errors were greater than 60 minutes on the high delay test days, while there were merely 3% and 2% on the other two types of test days. The reason for this difference was that severe delay flights destabilized the ATM system, which increased the challenge for prediction.

### Contribution of parameter models

**Fig 8:**
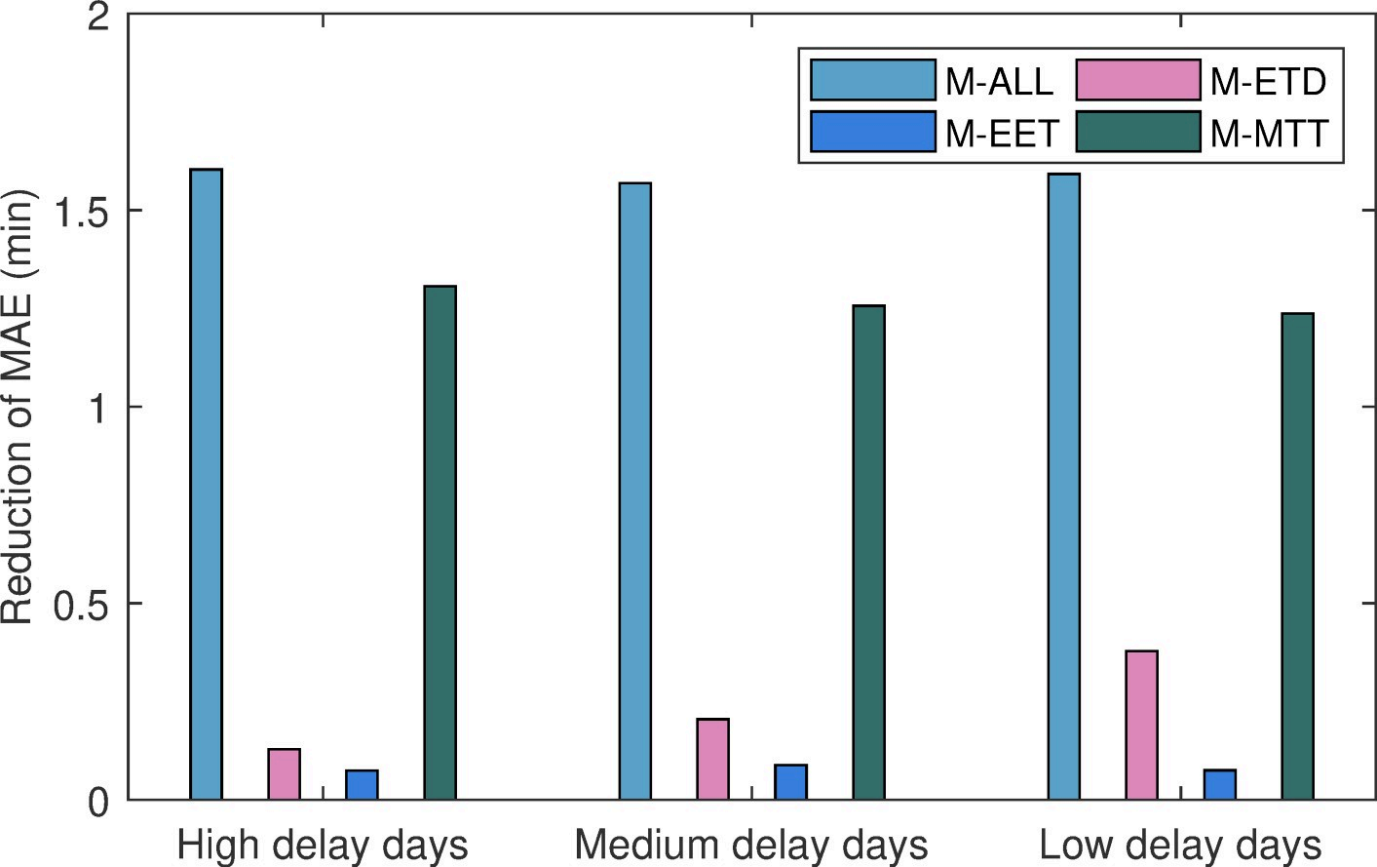
The contribution of the parameter methods.

The contribution of each parameter model was studied through the reduction of error, which was defined as the values of the MAE of the agent-based delay prediction model without introducing parameter models (the expected departure time model, the estimated elapsed time model, and the minimum turnaround time model) minus the error for the delay prediction model based on the parameter model(s) in a two-hour prediction horizon. The delay prediction models based on the parameter model(s) included the model considering all parameter models (M-ALL), the expected departure time model (M-EDT) only, the model considering the estimated elapsed time (M-EET) only, and the minimum turnaround time model (M-MTT) only. As illustrated by Fig 8, the reduction of the error of the delay prediction model using a single parameter model (M-EDT, and M-RED and MTT) was less than the reduction error of the M-ALL. This meant that our model was considerably effective with the combination of the PMs, and it provided the irreplaceable advantages that a single parameter model could not. We could also see from Fig 8 that the M-MTT displayed the largest error reduction compared with the other two models (M-ETD, M-EET) in three days. This meant that the minimum turnaround time (MTT) model had the largest contribution among the PMs. However, this feature also effectively explained the airline’s operating strategy to reduce the propagation of delays by setting the turnaround time. In contrast, the M-EET showed the smallest amount of error reduction in three tests. However, this also seemed reasonable since there could not be too many flights whose scheduled elapsed time had a very large difference with the schedule taking into account the limitations of aircraft fuel in the actual operation.

Moreover, we noticed that the expected departure time (EDT) model provided different contributions with the three types of test days, and it seemed to have a greater contribution to the low delay test days. One possible explanation for this was that the departure time of a flight was the result of the combined effect of the EDT and the airport capacity. Our weather-impacted airport capacity setting method reduced the contribution of the EDT model on high-delay days due to the decrease in airport capacity, while the EDT model might have been able to exert greater efficiency without capacity restrictions on low delay days. For example, a delayed flight that might have been caused by carrier factors would continue to be delayed for this reason for a while in the future. On high delay days, even though it had the ability to depart (for example, have already completed boarding), the lower airport capacity was more likely to be the ultimate limitation for its departure. However, on low delay days, due to the lack of the limitation of origin airport capacity, the continued delay in the future might only be contributed by the EDT. Therefore, it seemed that the impact on the capacity covered and impaired the function of the EDT on a high delay day. Overall, all of our parameter models more or less played a role, and their combination offered higher performance compared to a single parameter model.

### Effect of increasing forecast horizon

**Fig 9:**
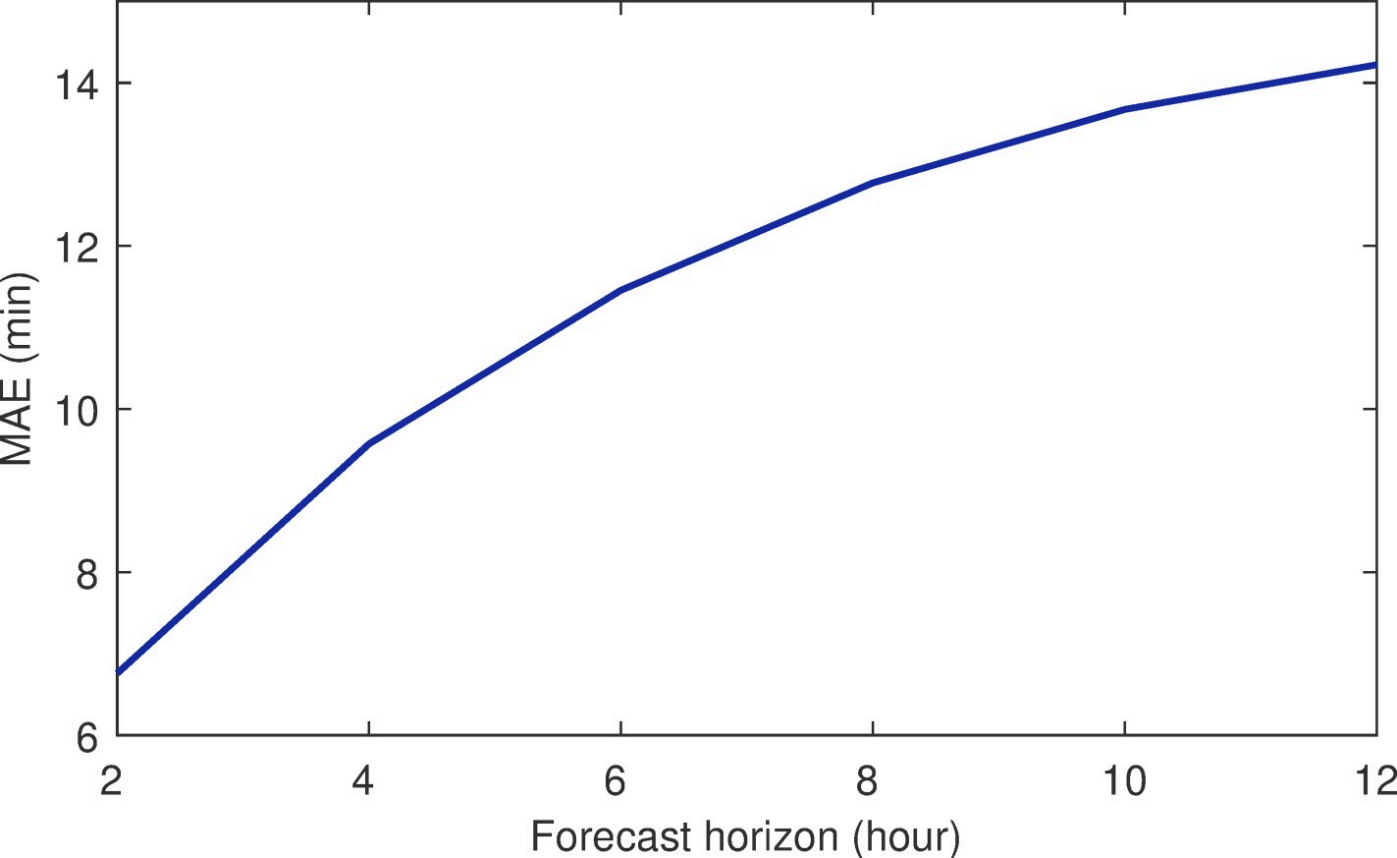
Forecast horizon vs. MAE.

The performance of the flight delay prediction model after increasing the prediction interval was studied further. As can be seen from Fig 9, the MAE and different forecast horizons were found to be 6.8 min (for a 2-h forecast horizon), 9.6 min (4-h forecast horizon), 11.5 min (6-h forecast horizon), and 14.2 min (12-h forecast horizon). As the prediction interval increased from 2 h to 4 h, 6 h, 8 h, 10 h, and 12 h, the real-time nature of obtaining the flight information decreased, and there was an error increase in the model initially. Subsequently, the error growth slowed down and tended to convergence. This demonstrated that our model could capture the changes in the environment when the forecast interval increased and the actual parameters (such as the airport capacity) were significantly different from before.

### Evaluating the model

Table 4 demonstrates the delay prediction accuracy of our model for the thirty test days with different delay levels. The MAE and the RMSE on the thirty test days were 6.8 min and 18.3 min, respectively. In addition to these two measures, the average classified accuracy with a threshold of 15 min for all the days was 89.5%. By comparing these values with the existing literature (Table 4) [28,36], it was apparent that our agent-based method incorporating with parameter estimation models has higher accuracy. In order to demonstrate the significance of the accuracy, the Mann-Whitney U-test was used to compare the positive and the negative differences between our model and the above existing literature. The Mann-Whitney U-test as a non-parametric test generally assumes that all the observations from both groups are independent of each other. Specifically, we verified the accuracy of significance compared to Guleria’s method as the outputs of Rebollo’s model are an aggregate delays [36]. The value of *p* is 4.9e-10, which indicates rejection of the null hypothesis at a 95 percent significance level, that is to say, there was a significant difference in the predicted values of flight delays generated by the two above methods. The result of the significance test implied that our agent-based method incorporating parameter estimation models was able to promote the delay prediction performance.

**Table 4:** The performance of existing literature.

Literature	Aggregate/individual delay	Data source	Classification accuracy
Rebollo and Balakrishnan [36]	aggregate (OD pairs)	the United States of America	81% (60 min threshold)
Guleria, et al. [28]	individual	Southeast Asia	80.7% (15 min threshold)
Our model	individual	the United States of America	89.5% (15 min threshold)

Apart from the improved accuracy, the runtime of the model could be greatly reduced to a great extent. Compared with the current methods and tools with up to several hours of runtime [20], merely a few minutes (less than 5 minutes) was needed with more than 20,000 flight agents and 360 airport agents running a modern computer (with 12 Intel Core 2.2 GHz processors and 16 GB RAM). Hence, the model could support decision-making in a more timely fashion in the tactical phase of ATFM.

## Conclusions and future work

Prior work has used simulation to investigate air traffic delay. However, these studies have not emphatically focused on the real-time parameters that significantly affect flight delay. Hence, it is difficult to apply these studies to flight delay prediction in the tactical phase. To fill this gap, we proposed a method combining simulation and data mining aiming to predict flight delays in the entire air traffic system in the future. In particular, an agent-based model was constructed, and several crucial time-varying parameters affected agent states obtained by data mining methods.

With thirty test days, the MAE and RMSE of the agent-based flight delay prediction model were 6.8 min and 18.3 min, respectively, with a 2-h forecast horizon. In addition, we achieved an accuracy of 89.5% with a threshold of 15 min. The model could provide considerable performance in virtually all cases with different delay levels. Although each parameter model had various contributions to the overall error reduction, they all played a role in the agent-based model. Additionally, the delay prediction model was robust to the increase in the prediction intervals, and it was capable of predicting flight delays in a large-scale air traffic network. The performance of our model provided the possibility for an air traffic manager to make real-time and effective initiatives to alleviate air traffic delays while considering the entire network operation information.

In order to further refine the model and improve the performance of the model, the impact of severe weather may be considered in future research. The weather condition was only introduced in the airport capacity setting. However, the estimated elapsed time (EET) was also impacted by enroute and airport weather, more or less. Furthermore, a more accurate airport capacity prediction model should be studied by integrating weather symbols such as thunderstorms and precipitation.
